# Supplementary material for: Quantum interference between dark-excitons and zone-edged acoustic phonons in few-layer WS2
Source: Nat Commun. 2023 Jan 6;14:88. doi: 10.1038/s41467-022-35714-3 (PMC9816112; doi:10.1038/s41467-022-35714-3)
Supplement: Supplementary file 1 — Supplementary Information [file 41467_2022_35714_MOESM1_ESM.pdf]

Supplementary Information for

"Quantum Interference between Dark-excitons and Zone-edged Acoustic Phonons in Few-layer WS_2"

Qing-Hai Tan[1,2,3,9], Yun-Mei Li[4,9], Jia-Min Lai[1,2], Yu-Jia Sun[1,2], Zhe Zhang[1,2], Feilong Song[1,2], Cedric Robert[5], Xavier Marie[5], Weibo, Gao[3,6,7], Ping-Heng Tan[1,2*], and Jun Zhang[1,2,8]*

[1]*State Key Laboratory of Superlattices and Microstructures, Institute of Semiconductors, Chinese Academy of Sciences, Beijing 100083, China*

[2]*Center of Materials Science and Optoelectronics Engineering, University of Chinese Academy of Sciences, Beijing 100049, China*

[3]*Division of Physics and Applied Physics, School of Physical and Mathematical Sciences, Nanyang Technological University, 637371, Singapore*

[4]*Department of Physics, Xiamen University, Xiamen, Xiamen 361005, China*

[5]*University of Toulouse, INSA-CNRS-UPS, LPCNO, 135 Av. Rangueil, 31077 Toulouse, France*

[6]*The Photonics Institute and Centre for Disruptive Photonic Technologies, Nanyang Technological University, 637371, Singapore*

[7]*Centre for Quantum Technologies, National University of Singapore, Singapore 117543, Singapore*

[8]*CAS Center of Excellence in Topological Quantum Computation, University of Chinese Academy of Sciences, Beijing 100049, China*

[9]*These authors contributed equally*

**Correspondence and requests for materials should be addressed to P. H. Tan (phtan@semi.ac.cn) and J. Z. (zhangjwill@semi.ac.cn)*

Section I, Supplementary Figures and Discussions

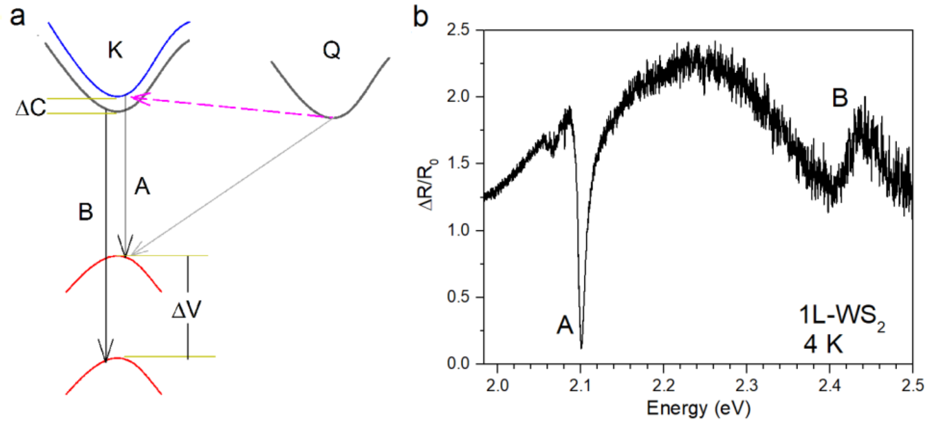

Supplementary Fig. S1 | The valance band splitting induced A and B excitons. a, The physical picture for valence band splitting induced A and B excitons transition. **b,** Reflectance contrast spectrum of 1L-WS_2 at 4 K.

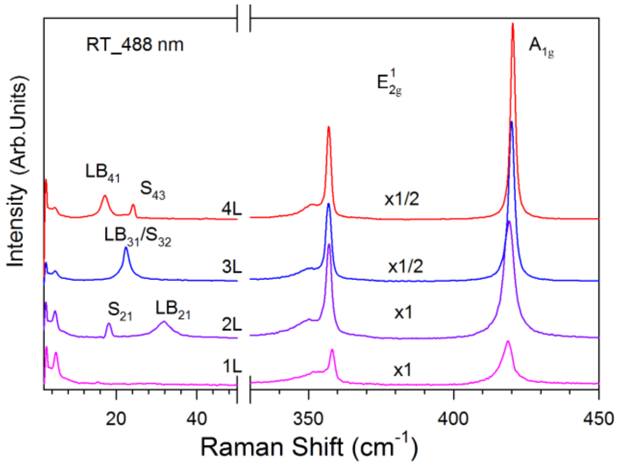

Supplementary Fig. S2 | Determining the layer number of WS_2 via ultralow frequency Raman spectroscopy. The Raman spectra of 1-4L WS_2 range from 5 cm^{-1} to 450 cm^{-1} at room temperature (RT). The frequencies of shear (S) modes and layer breathing (LB) modes are strictly dependent on the layer numbers of WS_2. The excitation wavelength is 488 nm here.

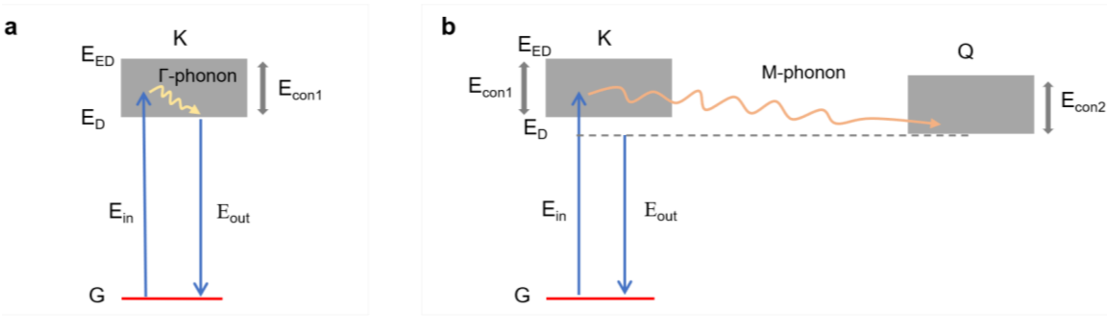

Supplementary Fig. S3 | Schematics of resonance-induced quantum interference processes. Γ point phonon-involved intravalley scattering (a) and M point phonon-involved intervalley scattering (b).

We can understand the quantum interference process as below. For Stokes Raman scattering, the relationship between incident light (E_{in}), scattering light (E_s), and phonon (E_{ph}) is $\omega_{in} = \omega_s + \omega_{ph}$. Meanwhile, for simplicity, the relationship between exciton-accumulated dark level (E_{ED}), pure dark level (E_D), and continuum bandwidth formed by dark excitons (E_{con}) is $E_{ED} = E_D + E_{con}$. Here the formation of the continuum states is a result of the accumulation of dark exciton both at K and Q valley thanks to their long lifetime feature when considering the time scale of exciton-phonon interactions and density of states in two-dimensional (2D) system, as discussed in the main text. We should note that there are no continuum states below the E_D. For resonant Raman scattering in our case, the energy of incident light (E_{in}) (scattering light (E_s)) is in resonance with the exciton state E_{ED} (E_D) instead of E_{ph}. As a result, here the Fano resonance arises from phonons and the continuum (E_{con}) state of the dark exciton, as shown in Fig. S3.

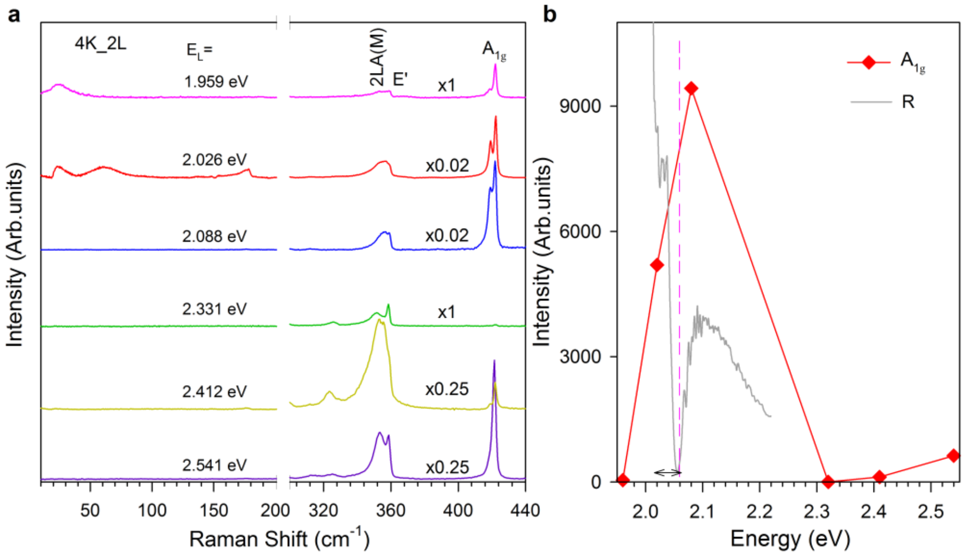

Supplementary Fig. S4 | Excitation energy dependence of Raman spectra of 2L WS_2. a, The full Raman spectra of 2L-WS_2 range from 5 cm^{-1} to 440 cm^{-1} at 4 K. **b,** The integrated intensity of A_{1g} mode as a function of excitation energies. The inset gray solid line shows the reflection spectrum of 2L-WS_2.

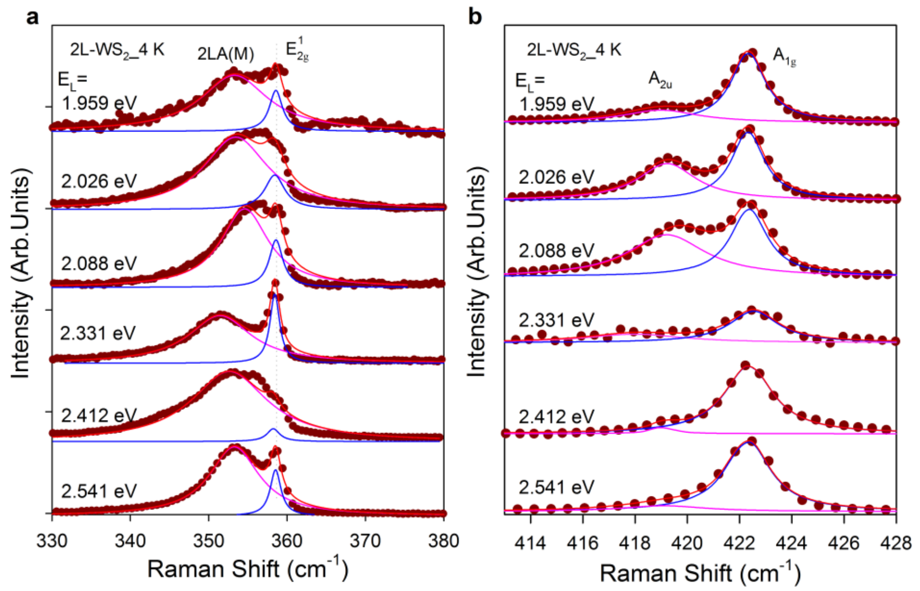

Supplementary Fig. S5 | Excitation energy dependence of Raman spectra of 2L WS_2. The Lorentzian fitting results 2LA(M), E^1_{2g} mode (a) and A_{1g} mode (b) of 2L-WS_2 with different excitation wavelengths. The A_{1g} mode shows a clear Davydov splitting when excitation energies are close to A exciton.

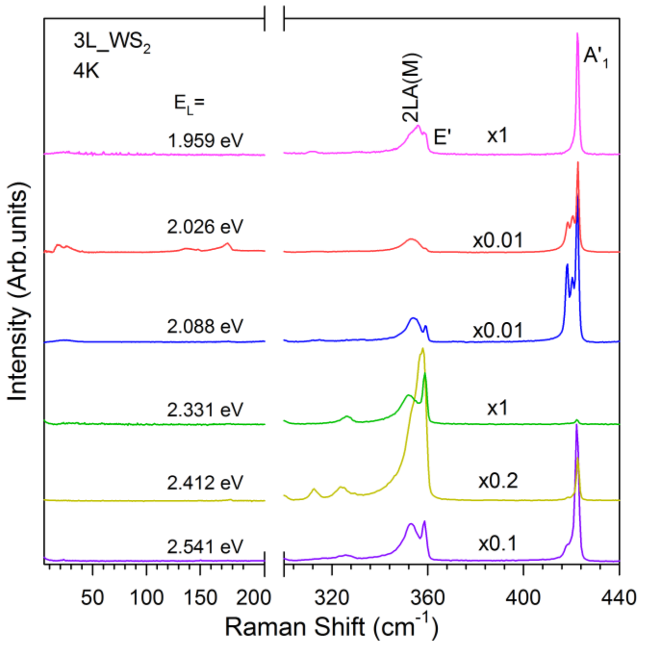

Supplementary Fig. S6 | Excitation energy dependence of Raman spectra of 3L WS_2. The full Raman spectra of 3L-WS_2 range from 5 cm^{-1} to 440 cm^{-1} at 4 K.

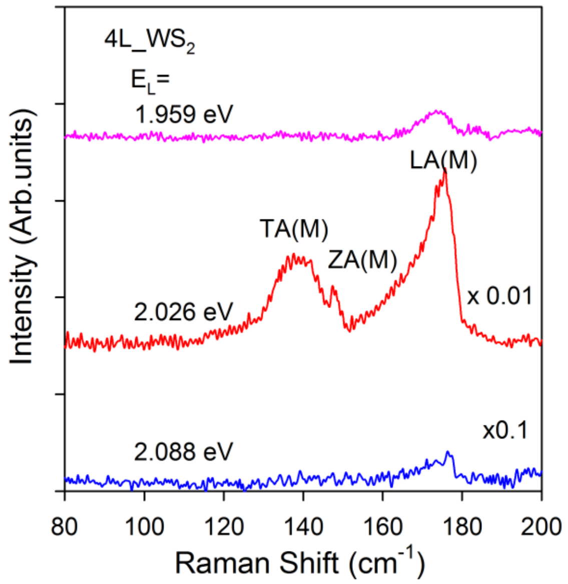

Supplementary Fig. S7 | Excitation energy dependence of Raman spectra of 4L WS_2 at 4 K.

By fitting and analyzing the high-frequency Raman spectra of few-layer WS_2, we found that there are no obvious peak broadening or asymmetry lineshape for 2LA(M), $E'(E^1_{2g})$ and $A'_{1g}\,(A'_1)$ modes, as shown in Fig. S4 to S7. It suggests that there are no Fano resonance for $E'(E^1_{2g})$ and $A'_{1g}\,(A'_1)$ phonon modes (the optical phonon modes at Γ point). The vibration of these modes is the relative motion inside the primitive cell atoms. Usually, the treatment of the interactions between electrons and these phonon modes should employ the polaron-related theory, different from this work's physical picture. Probably this is why we do not observe the quantum interference phenomenon for high frequency optical branch phonon modes.

We note that the energy of 594 nm excitation (2.09 eV) wavelength is slightly higher than the X_0 in few-layer WS_2. In that case, the high-frequency A_{1g} mode is greatly enhanced, implying that the out-of-plane A_{1g} mode strongly couples to the bright A excitons. This can be understood that the propagation (polarization) direction of bright A exciton is out-of-plane (in-plane), which can effectively couple with out-of-plane A_{1g} phonons.

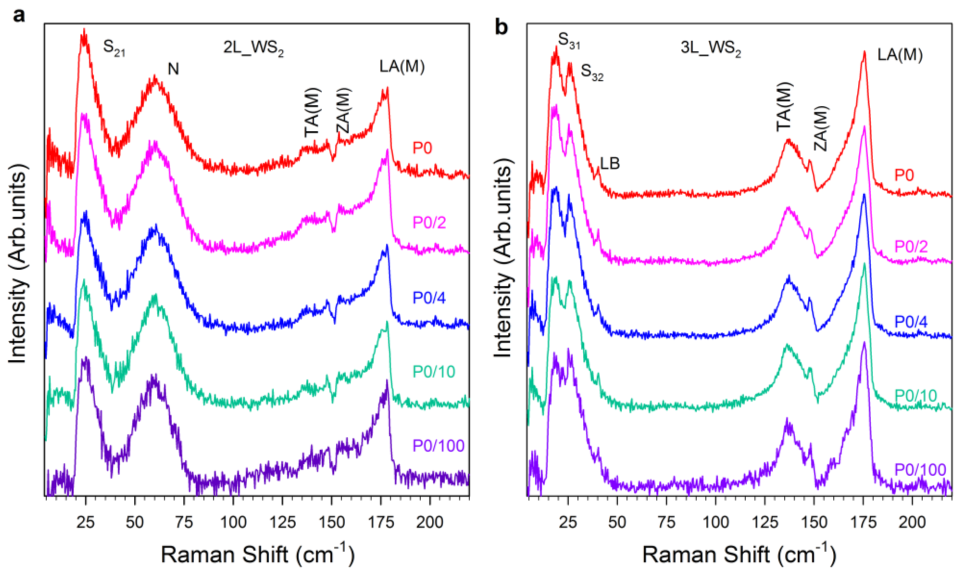

Supplementary Fig. S8 | Excitation power dependence of Raman spectra of 2L and 3L WS_2. a Power-dependent Raman spectra of LA(M) and TA(M) phonon modes in 2L- and 3L WS_2 at 4 K, respectively. Here the intensities are normalized to the LA(M) modes. The laser power P0 is around 0.6 mW. The Fano profile almost keeps unchanged, and the intensity increases linearly with laser power, implying these processes correspond to first-order Raman scattering.

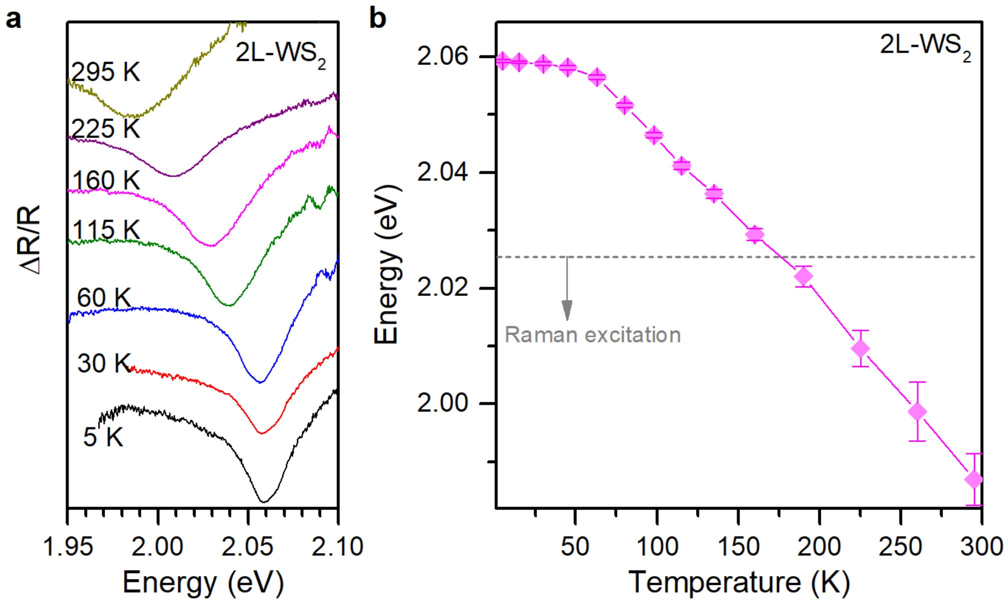

Supplementary Fig. S9 | Temperature dependence of reflectance spectra of 2L WS_2. a, The temperature dependence of reflectance contrast spectra of 2L WS_2. **b,** The bright A exciton energies as a function of temperature. The dotted gray line indicates the 612 nm laser for Raman excitation in the Fig. 4 in main text.

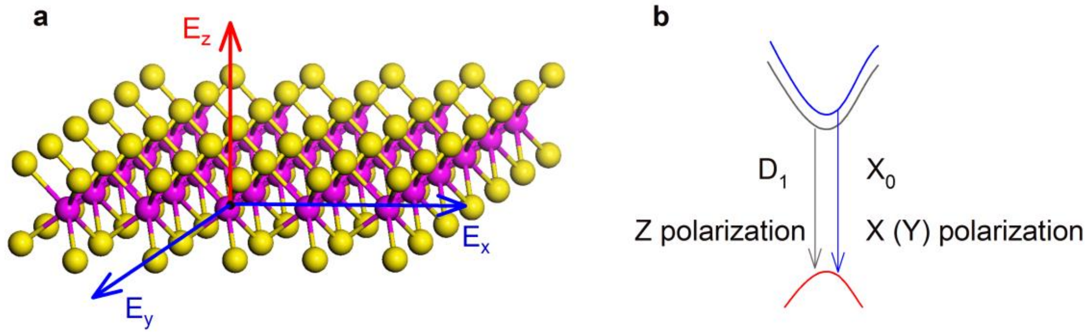

Supplementary Fig. S10 | Schematic of bright and dark exciton polarization. a, Side of the monolayer WS_2 with E_X, E_Y and E_Z axis. **b,** The polarization direction of bright (X_0) and dark (D_1) A exciton.

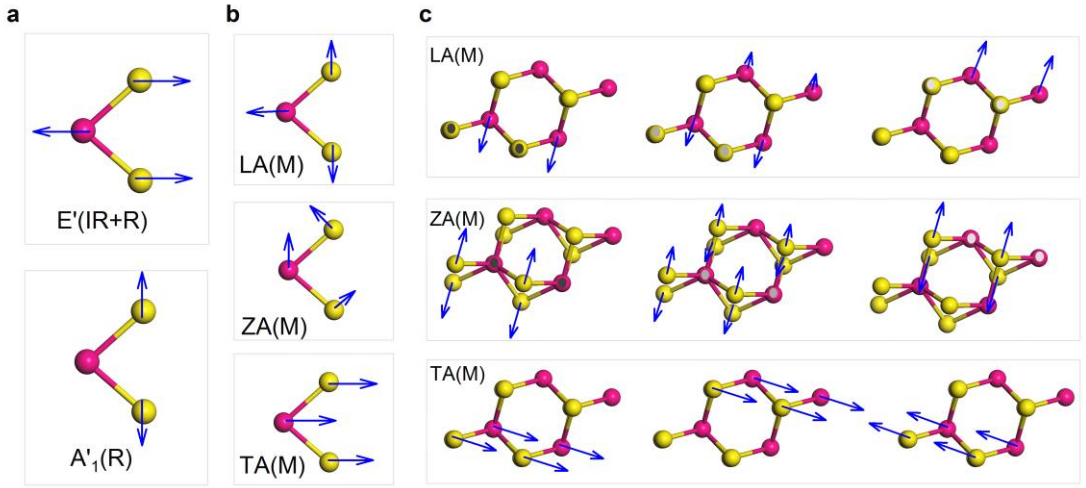

Supplementary Fig. S11 | Schematic of vibration way of phonon modes. a,b, Side view of the schematic of vibration way of *E*' and A'_1 modes, as well as the vibration way of LA (M), ZA(M) and TA(M) modes. **c,** Detailed evolution of vibration schematic of LA (M), ZA(M) and TA(M) modes.

Section II, Supplementary table

Table 1 | The fitting parameters *q* with the fitting errors of Fano profile of phonon modes in 2L and 3L-WS_2 at 4 K.

	Shear mode	ZA(M)	LA(M)
2L-WS_2	2.9 ± 0.3	-0.14±0.03	-3.2±0.2
3L-WS_2	2.6±0.2	-3.1±0.4	-4 ± 0.2

Section III, Symmetry Analysis of phonon modes and excitons

Phonon modes at Γ point and M point:

For the shear (S) modes at Γ point, the phonon symmetry is E_{2g}^{2}. The corresponding Raman tensor is doubly degenerate.

$$R(E_{2g}^{2}):\begin{pmatrix}0 & d & 0\\ d & 0 & 0\\ 0 & 0 & 0\end{pmatrix},\begin{pmatrix}d & 0 & 0\\ 0 & -d & 0\\ 0 & 0 & 0\end{pmatrix}.$$

For the layer breathing (LB) modes at Γ point, the phonon symmetry is A'_1 (or A_{1g}).

$$R(A_{1g}):\begin{pmatrix}a & 0 & 0\\ 0 & a & 0\\ 0 & 0 & b\end{pmatrix}$$

The symmetry of M-point in the Brillouin zone is D_{2h}. There are four symmetries A_g, B_{1g}, B_{2g}, B_{3g}. The symmetry for LA(M) phonon is A_g, for TA(M) is B_{1g}, for ZA(M) is B_{3g}. The corresponding Raman tensors are

$$R(A_g)=\begin{pmatrix}a & 0 & 0\\ 0 & b & 0\\ 0 & 0 & c\end{pmatrix},\quad R(B_{1g})=\begin{pmatrix}0 & d & 0\\ d & 0 & 0\\ 0 & 0 & 0\end{pmatrix},\quad R(B_{3g})=\begin{pmatrix}0 & 0 & 0\\ 0 & 0 & f\\ 0 & f & 0\end{pmatrix}.$$

Under the resonant excitation with dark A exciton, the Raman selection rules are determined by the exciton-phonon interaction, rather than by the Raman tensor of phonon modes [1], as a result, these Raman inactive modes also can be observed.

Exciton symmetry:

The exciton symmetry is composed of symmetry of the envelop function (internal orbital symmetry) and the electron (conduction band) and hole (valence band) symmetry. The representation can be given by $\Gamma = \Gamma_{env} \otimes \Gamma_c \otimes \Gamma_v^*$ [2]. Here we only need to consider the symmetry of *1s* state in our case.

For bright A exciton, the symmetry is $\Gamma^{1s} = \Gamma_{11} \otimes \Gamma_7^* = \Gamma_2$ in C_{3h} symmetry at K point. The polarization of bright A exciton is in-plane (X or Y polarization) [2-4].

For dark A exciton, the symmetry is $\Gamma^{1s} = \Gamma_9 \otimes \Gamma_7^* = \Gamma_9 \otimes \Gamma_8 = \Gamma_4$ in C_{3h} symmetry at K point. The polarization of dark A exciton is out-of-plane (Z

polarization, corresponding to a z-dipole transition), which is in-plane dipole transition forbidden for normal incidence configuration [2-4]. We should note that the dark A exciton is slightly mixed with bright A exciton, which could be brightened by a large in-plane magnetic field [5-7].

For dark B exciton, the symmetry is $\Gamma^{1s} = \Gamma_{11} \otimes \Gamma_8^* = \Gamma_{11} \otimes \Gamma_7 = \Gamma_6$ in C_{3h} symmetry at K point. Thus the dark B excitons have strictly zero oscillator strength. Meanwhile, the dark B exciton state is the upper band one (Fig. S1). The forming of a continuum state by exciton population is more difficult than that for the dark A exciton. As a result, under such resonance excitation conditions, the Fano resonance of shear modes basically vanishes, and the Fano resonance of zone-edged acoustic modes at the M point is greatly weakened.

For the dark exciton with finite momentum, the electron at Q valley holds σ symmetry while the hole at K valley holds a C_{3h} symmetry. The dark excitons are momentum forbidden.

The resonance situation with dark B excitons

We note that a weak Fano intensity of ZA(M) and LA(M) can be resolved with 514 nm excitation. This result can be understood by considering that this excitation energy is close to B exciton. Hence, the scattering picture that we mentioned above (see Fig. S1) results in the observed ZA(M) and LA(M). However, the nature of dark B exciton states (upper band and zero oscillator strength [3] and the above optical bandgap excitation significantly weaken such process.

References

1. Tan, Q.-H. *et al*. Breakdown of Raman selection rules by fröhlich interaction in few-layer WS_2. *Nano Research* **14**, 239–244 (2021).

2. Echeverry, J. P., Urbaszek, B., Amand, T., Marie, X. & Gerber, I. C. Splitting between bright and dark excitons in transition metal dichalcogenide monolayers. *Phys. Rev. B* **93**, 121107 (2016).

3. Wang, G. *et al*. In-plane propagation of light in transition metal dichalcogenide monolayers: Optical selection rules. *Phys. Rev. Lett*. **119**, 047401 (2017).

4. K.-D. Park, T. Jiang, G. Clark, X. Xu, and M. B. Raschke, Radiative control of dark excitons at room temperature by nano-optical antenna-tip Purcell effect, *Nature Nanotechnol*. **13**, 59 (2018).

5. Molas, M. R. et al. Brightening of dark excitons in monolayers of semiconducting transition metal dichalcogenides. *2D Mater*. **4**, 021003 (2017).

6. Zhang, X.-X. et al. Magnetic brightening and control of dark excitons in monolayer WSe2. *Nat. Nanotechnol*. **12**, 883 (2017).

7. Zhou, Y. *et al*. Probing dark excitons in atomically thin semiconductors via near-field coupling to surface plasmon polaritons. *Nat. Nanotechnol*. **12**, 856 (2017).
